# Structural Modification of Selected Essential Oil Components for Potential Anticancer Applications: A Review

**DOI:** 10.3390/ph19030427

**Published:** 2026-03-05

**Authors:** Vuyolwethu Khwaza, Vuyani Maqanda

**Affiliations:** Department of Earth and Chemical Sciences, Chemistry Discipline, University of Fort Hare, Alice 5700, Eastern Cape, South Africa; vmaqanda@ufh.ac.za

**Keywords:** phytochemicals, monoterpenes, derivatives, structural modification, analogues, anticancer

## Abstract

Monoterpenes (thymol, carvacrol, menthol) and phenylpropanoids (eugenol and cinnamaldehyde) and their related derivatives are naturally occurring bioactive compounds found in essential oils (EOs) and have attracted considerable interest as anticancer agents; however, their direct therapeutic use in cancer treatment is often limited by factors such as low bioavailability, moderate potency, and lack of target specificity. Recent studies have demonstrated that rational structural modification of these EO scaffolds can substantially enhance their anticancer potential. This review critically evaluates the different structural modification strategies applied to EO components, including pharmacophore hybridization, heterocycle incorporation (e.g., triazoles, oxadiazoles, chalcones), esterification, halogenation, metal complexation, and nanoparticle conjugation. The review compares these approaches across the selected EO components, highlighting their impact on anticancer potency, and mechanistic relevance. However, the current evidence base is heterogeneous, with considerable variability in experimental conditions, selectivity assessments, and reliance on in vitro or in silico findings, which limits direct cross-study comparisons and translational interpretation. Overall, structural modification of EO components represents a promising strategy for generating novel anticancer lead compounds, but future progress will depend on standardized biological evaluation, rigorous in vivo validation, and comprehensive pharmacokinetic and toxicity profiling to realistically define their clinical potential.

## 1. Introduction

Cancer continues to be a major global health challenge, ranking among the leading causes of morbidity and mortality. According to the US National Cancer Institute, in 2022, nearly 20 million new cancer cases and 9.7 million related deaths were reported worldwide [1]. Projections indicate that by 2050, annual new cancer cases could reach 33 million, with approximately 18.2 million deaths attributed to the disease. Despite significant advances in conventional therapeutic strategies, including chemotherapy, radiotherapy, and immunotherapy, limitations such as drug resistance, toxicity, and poor selectivity toward malignant cells continue to hinder effective treatment [2,3,4]. This has intensified the search for novel, safer, and more effective anticancer agents, with natural products emerging as a valuable source of structurally diverse and biologically active compounds.

**Figure 1:**
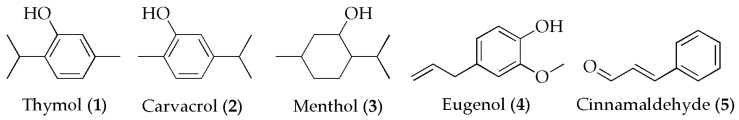
Selected EO components: monoterpenes (thymol **1**, carvacrol **2**, Menthol **3**) and phenylpropanoids (eugenol **4** and cinnamaldehyde **5**).

Among natural products, monoterpenes and phenylpropanoids represent large and diverse classes of secondary metabolites commonly found in EOs of aromatic plants [5,6,7]. Within these groups, EO components such as thymol, carvacrol, menthol, eugenol, cinnamaldehyde (Figure 1) and related derivatives have gained significant attention due to their wide spectrum of biological activities, including antimicrobial, antioxidant, anti-inflammatory, and anticancer effects [6,8,9,10]. Their relatively simple chemical structures and functional versatility make them attractive scaffolds for medicinal chemistry research.

However, the direct application of these EO components in cancer therapy is constrained by certain drawbacks, including low water solubility, poor bioavailability, limited metabolic stability, and modest potency [11,12,13]. To overcome these challenges, extensive efforts have been devoted to the structural modification of these natural molecules, with the aim of improving their pharmacological properties and enhancing their anticancer activity. Strategies such as esterification, etherification, acylation, metal complexation, and hybridization with heterocyclic moieties have yielded novel derivatives with improved cytotoxicity, selectivity, and mechanistic diversity against various cancer cell lines.

This review provides a comprehensive overview of the recent advances in the structural modification of the selected OE components, emphasizing their potential as anticancer agents. It highlights the chemical strategies employed, summarizes reported anticancer activities, and discusses underlying molecular mechanisms of action. These compounds are among the most abundant and extensively studied constituents of EO and have repeatedly demonstrated intrinsic anticancer potential across diverse cellular models. Importantly, each scaffold contains chemically versatile functional groups (e.g., phenolic hydroxyls, allylic side chains, or aldehyde moieties) that permit a wide range of rational derivatization strategies, including hybridization, heterocycle incorporation, esterification, and nanoparticle conjugation. The relatively rich body of literature reporting both structural modifications and biological evaluation for these scaffolds allows meaningful comparative analysis of how different chemical modification approaches influence potency, selectivity, and mechanism of action. Therefore, rather than attempting an exhaustive survey of all EO components, this review adopts a focused and analytically driven scope centered on these five well-characterized and synthetically tractable scaffolds to provide clearer guidance for future anticancer design efforts.

## 2. Methodology

This review focuses on five selected EO-derived scaffolds (thymol, carvacrol, menthol, eugenol, and cinnamaldehyde) (Figure 1) selected based on their high prevalence in medicinal chemistry literature, chemical modifiability, documented anticancer bioactivity, and translational relevance. A structured literature search was conducted using databases including Scopus, Web of Science, PubMed, and Google Scholar, covering studies published from 2021 to 2025. Inclusion criteria comprised peer-reviewed articles reporting the synthesis and biological evaluation of structurally modified derivatives of the selected EO scaffolds with anticancer activity. Studies limited to unmodified parent compounds or lacking quantitative biological evaluation were excluded. Emphasis was placed on comparative analysis of modification strategies.

## 3. Overview of Monoterpenes and Phenylpropanoids

Monoterpenes and phenylpropanoids are two major classes of naturally occurring bioactive compounds widely found in EOs and known for their diverse pharmacological properties, including antimicrobial [14,15,16], antioxidant [17], and anti-inflammatory [18], analgesic [19], and anticancer properties [11,20,21,22]. Their occurrence in nature is linked to the plants’ defense mechanisms against microbial infections, herbivory, and oxidative stress [5]. Monoterpenes are primarily composed of two isoprene (C_5_) units, forming a C_10_ backbone that can exist as acyclic, monocyclic, or bicyclic structures. Common examples such as thymol, carvacrol, and menthol often contain additional functional groups, including phenolic hydroxyls or alcohol moieties, which contribute to their chemical reactivity and biological activity [23,24]. Their phenolic hydroxyl or alcohol moieties enable them to act as radical scavengers, thereby mitigating oxidative damage associated with chronic diseases, including cancer [25,26]. These compounds are predominantly found in EOs extracted from plants such as thyme (*Thymus vulgaris*), oregano (*Origanum vulgare*), clove (*Syzygium aromaticum*), and peppermint (*Mentha piperita*) [8]. Due to their natural abundance, low toxicity, and structural simplicity, phenolic monoterpenes are considered promising lead compounds in drug discovery and development.

In contrast, phenylpropanoids are derived from the amino acid phenylalanine through the shikimate pathway and possess a C_6_–C_3_ aromatic skeleton [27,28]. Key examples, including eugenol and cinnamaldehyde, contain functional groups such as phenols, methoxy groups, and aldehydes that influence their pharmacological properties [29,30]. These compounds are typically isolated from various parts of aromatic and medicinal plants, including leaves, flowers, seeds, and bark, of plant families such as *Lamiaceae* [31,32,33], *Myrtaceae* [34,35], and *Zingiberaceae* [36,37].

Despite their promising pharmacological potential, unmodified monoterpenes and phenylpropanoids face several limitations that hinder their clinical application in cancer therapy. These include poor water solubility, high volatility, and rapid metabolic degradation, which collectively result in low bioavailability and short half-life in systemic circulation [17]. Additionally, their non-selective cytotoxicity at higher concentrations may pose safety concerns [38]. The limited stability of these compounds under physiological conditions further restricts their therapeutic efficacy [8]. The structural diversity and widespread natural occurrence of monoterpenes and phenylpropanoids make them attractive scaffolds for chemical modification, enabling the development of derivatives with enhanced therapeutic potential, particularly in anticancer applications.

The following sections highlight different strategies used in structural modification of the selected EO components and summarize key advances in generating derivatives with enhanced anticancer potential. Emphasis is placed on the chemical rationale behind each modification, representative examples from recent literature, and insights into structure–activity relationships (SARs) that guide the development of more potent and selective anticancer candidates.

## 4. Derivatives of Selected EO Components and Their Anticancer Potential

### 4.1. Carvacrol/Thymol

Carvacrol and thymol are structurally related phenolic monoterpenes abundantly present in EOs from *Origanum* and *Thymus* species and are well recognized for their broad spectrum of biological activities [39,40]. In recent years, both compounds have attracted growing interest as promising anticancer scaffolds due to their capacity to modulate multiple cancer-related pathways, including cell cycle regulation, apoptosis induction, oxidative stress, and inflammation [41,42]. However, their clinical translation is limited by moderate potency and bioavailability [43]. To overcome these limitations, extensive structural modifications of carvacrol and thymol have been pursued. These derivatization strategies have yielded carvacrol- and thymol-based compounds with enhanced cytotoxic potency, improved selectivity toward cancer cells, and favorable drug-like properties.

**Figure 2:**
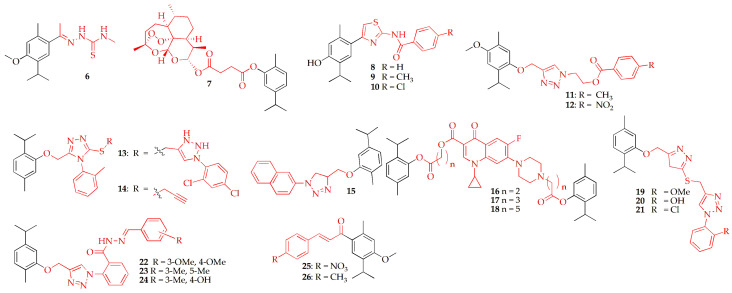
Thymol/carvacrol-based derivatives synthesized via hybridization strategies.

Laamari et al. [44] synthesized a series of novel 1,3,4-thiadiazole, 1,3-thiazole, and 1,3-thiazolidin-4-one derivatives linked to thymol by cyclizing the corresponding thiosemicarbazones with reagents such as acetic anhydride, ethyl bromoacetate, dimethyl acetylene dicarboxylate, and phenacyl bromides. The resulting thiosemicarbazones and their heterocyclic derivatives were evaluated for in vitro cytotoxicity against four human cancer cell lines: breast adenocarcinoma (MCF-7, MDA-MB-231), lung carcinoma (A-549), and fibrosarcoma (HT-1080), with doxorubicin serving as a reference. Most compounds exhibited moderate to high cytotoxic activity, with thiosemicarbazone (**6**) (Figure 2, Table 1) showing particularly strong effects against HT-1080 and A-549 cells, with IC_50_ values of 7.10 ± 0.32 µM and 14.40 ± 0.36 µM, respectively. Further studies demonstrated that compound **6** induced fourteen times more apoptosis at 10 μM compared to the control, and caused G2/M phase cell cycle arrest along with caspase-dependent apoptosis, as evidenced by flow cytometry in both HT-1080 and A-549 cell lines.

Mbese et al. [45] developed a series of carvacrol-based hybrid compounds exhibiting both antibacterial and anticancer activities. The compounds were synthesized via esterification reactions between carvacrol and known pharmacophores at room temperature and were thoroughly characterized using ^1^H NMR, ^13^C NMR, and UHPLC-HRMS. In vitro cytotoxicity evaluation revealed that compound **7** (Figure 2, Table 1), in which carvacrol was linked to artesunate, induced significant cytotoxic effects across all tested cancer cell lines, including MDA (16.57 ± 1.14 µM), MCF-7 (0.47 ± 1.14 µM), and DU145 (16.25 ± 1.08 µM), as well as in normal breast cells, MCF-12A (0.75 ± 1.30 µM).

Bouribab et al. [46] investigated the anticancer potential of newly synthesized amino-thiazole-based thymol derivatives using advanced computational approaches. Density functional theory (DFT/B3LYP/6-31G(d,p)) was applied to examine their molecular structures, stability, reactivity, and key quantum chemical properties. Compound **8** (Figure 2, Table 1) displayed a high energy gap (3.886 eV), indicating strong stability and low reactivity. Molecular docking studies revealed strong interactions of compounds **9** and **10** (Figure 2, Table 1) with tyrosine kinase targets, particularly EGFR and PDGFR, with binding energies of −8.9 and −9.3 kcal/mol, respectively. These results were further supported by molecular dynamics simulations. Additionally, ADMET predictions indicated good drug-likeness, compliance with Lipinski’s rules, and promising oral bioavailability.

Laamari et al. [47] synthesized and characterized a new series of *p*-methoxythymol-linked 1,2,3-triazole derivatives, with their structures confirmed by HRMS and detailed ^1^H and ^13^C NMR analysis. Network pharmacology identified phosphatidylinositol-3-kinase (PIK3CA) as a primary molecular target, and ADMET screening, along with molecular docking studies, indicated favorable drug-like properties. Among these derivatives, compound **11** (*p*-methyl) and **12** (*p*-nitro) (Figure 2, Table 1) showed the best docking affinities (−8.4 and −7.8 kcal/mol, respectively) and formed strong interactions with key residues ARG818 and ASN170. Molecular dynamics simulations confirmed stable ligand–protein complexes over 100 ns. Overall, compounds **11** and **12** emerged as promising lead candidates for targeting PIK3CA in breast cancer therapy.

Alam et al. [48] synthesized new thymol–triazole hybrids using the Click Chemistry approach and evaluated their anticancer activity. In this study thymol was used as the starting material to prepare intermediates compounds (via reported methods [49]), after which the intermediates compound was S-alkylated with phenyl thiocyanate in ethanol/triethylamine (TEA) to give triazole hybrid, propargylated with 3-bromo-prop-1-yne in acetone/K_2_CO_3_ to yield compound **13** (Figure 2, Table 1), and finally subjected to CuSO_4_/sodium ascorbate-catalyzed click reaction with various aryl azides in tBuOH/H_2_O (1:1) to afford thymol–triazole hybrids in good yields. These hybrids showed notable antiproliferative activity, particularly against HepG2 adenocarcinoma cells. Among them, compound **13** demonstrated the strongest cytotoxic effects against MCF-7, HepG2, and HCT-116 cell lines with IC_50_ values of 3.52, 1.02, and 4.12 µM, respectively, and potent thymidylate synthase (TS) inhibition (IC_50_ = 0.21 µM). It also induced G_2_ phase cell cycle arrest, increasing the cell population from 1.14% to 84.07% and causing 96% late apoptosis. Molecular docking study revealed binding interactions similar to 5-fluorouracil, supporting its potential as a powerful TS inhibitor for cancer therapy. Compound **14** (Figure 2) with IC_50_ of 4.21 and 5.01 was also found to be better against MCF-7 and HepG2, respectively, when compared to doxorubicin.

Enneiymy et al. [50] successfully synthesized a series of novel triazole–carvacrol hybrid molecules using a catalytic method, achieving good to excellent yields. This method commenced with the preparation of 4-isopropyl-1-methyl-2-(prop-2-ynyloxy)benzene and the corresponding aromatic azides, following the procedures reported by Aneja et al. [51] and Zarei et al. [52], respectively. The target hybrid compounds were synthesized via a Cu(I)-catalyzed azide–alkyne cycloaddition (click reaction) between propargyl carvacrol and 2-azidoethan-1-ol in an ethanol–water system at room temperature, followed by standard work-up and silica gel column chromatography. The synthesized compounds were comprehensively characterized, and their anticancer potential was further evaluated in silico. Among the series, compound **15** (Figure 2, Table 1) emerged as the most potent inhibitor of the enzymes EGFR, BRAF^V600E, and tubulin, exhibiting lower binding energies (calculated using AutoDock 4.2) compared to the reference drugs encorafenib, colchicine, and sorafenib. Molecular dynamics simulations further confirmed the stability of compound **15** within the ligand–protein complexes, maintaining consistent structural integrity throughout a 100-ns simulation. These findings highlight compound **15** as a promising candidate for further development as an anticancer agent.

Szostek et al. [53] synthesized sixteen new ciprofloxacin derivatives fused with menthol and thymol using diverse carboxylic linkers. Thymol-based ciprofloxacin hybrid derivatives were synthesized by reacting ciprofloxacin with the corresponding thymol ester in DMF in the presence of NaHCO_3_ at 70 °C, followed by acidic work-up, extraction, and purification by silica gel column chromatography. In silico analysis evaluated their lipophilicity, and in vitro cytotoxicity was assessed using the MTT assay. Thymol-based compounds (**16**–**18**) (Figure 2, Table 1) exhibited strong anticancer activity while remaining non-toxic, identifying them as leading compounds.

Almalki et al. [48] synthesized 1,2,3-triazole-linked thymol–oxadiazole hybrids via click chemistry using aromatic azides and an *S*-propargylated 1,3,4-oxadiazole intermediate derived from thymol through sequential esterification, cyclization, propargylation, and oxidation steps, with structures confirmed by analytical spectroscopic techniques. Among these compounds, **19**–**21** (Figure 2, Table 1) demonstrated strong anticancer activity. Compound **20** showed the highest potency against MCF-7, HCT-116, and HepG2 cells (IC_50_ 1.1–1.4 μM), surpassing doxorubicin and 5-fluorouracil. Compounds **19** and **20** were also highly active, with ortho-substituted derivatives consistently outperforming meta and para analogues. The anticancer effects were attributed to TS inhibition, with several compounds showing stronger TS inhibition (IC_50_ 1.95–4.24 μM) than pemetrexed (7.26 μM), supporting their potential as potent anticancer leads.

Çakır et al. [54] synthesized carvacrol-based arylidene hydrazide derivatives from anthranilic acid methyl ester. These compounds were synthesized via diazotization–azidation of methyl anthranilate, CuAAC click coupling of the resulting azide with carvacrol-derived propargyl ether to form a triazole, followed by ester-to-hydrazide conversion and final condensation with substituted benzaldehydes under acidic conditions. The compounds were evaluated for cytotoxicity against A549 (lung cancer) and BEAS-2B (normal lung) cells, with compounds **22**–**24** (Figure 2, Table 1) showing potent selective anticancer activity. In silico studies, including molecular docking and 100 ns molecular dynamics simulations, revealed key interactions with EGFR (Lys-745, Phe-856) and BRAF (Lys-483), while ADME predictions indicated favorable pharmacokinetic properties, particularly for compound **23**, highlighting these derivatives as promising selective anticancer agents.

Laamari et al. [55] reported an efficient synthesis of chalcone derivatives derived from natural thymol. These derivatives were synthesized from thymol via sequential alkylation with iodomethane, Friedel–Crafts acetylation to afford the corresponding acetyl intermediate, and subsequent base-catalyzed Knoevenagel condensation with aromatic aldehydes, yielding the target compounds in good yields. All synthesized compounds exhibited favorable physicochemical properties and ADMET profiles. Molecular docking studies demonstrated strong interactions with the EGFR target protein, with compound **25** (*p*-nitro) and **26** (*p*-methyl) (Figure 2, Table 1) showing the most favorable docking scores and low-energy conformations (−8.02 kcal/mol and −7.42 kcal/mol, respectively) compared to the reference drug gefitinib. Molecular dynamics simulations further confirmed the stability of compounds **25** and **26** within the ligand–protein complexes over 100 ns, with acceptable RMSF values indicating structural integrity and stable protein–ligand interactions. These findings highlight compounds **25** and **26** as promising lead candidates for EGFR-targeted anticancer therapy.

**Figure 3:**
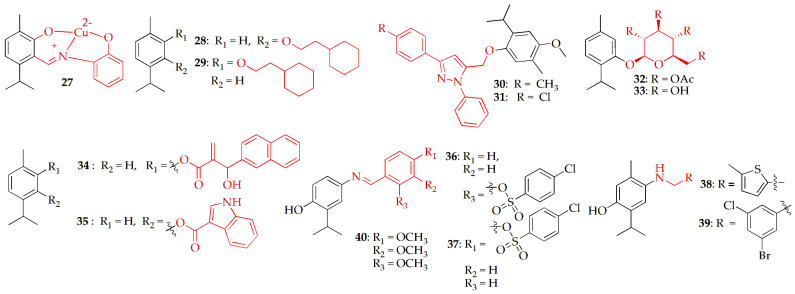
Thymol/carvacrol-based derivatives synthesized via metal complexation (**27**), esterification (**34** & **35**), etherification (**28**–**33**), and Schiff base formation (**36**–**40**) strategies.

Bansal et al. [56] developed derivatives of carvacrol such as carvacrol aldehyde, a Schiff base, and a copper–Schiff base complex using a standardized synthetic method. The synthetic route followed a three-step process: (i) formylation of carvacrol to generate carvacrol aldehyde, (ii) Schiff base condensation with 2-aminophenol, and (iii) complexation with Cu(II) to yield a crystalline Cu–Schiff base complex, illustrated as compound **27** in Figure 3. The derivative was tested for their in vitro cytotoxic effects against several cancer cell lines, including human lung cancer (A549) and human fibroblast (BALB-3T3) (see Table 1). Results indicated that the copper–Schiff base complex effectively suppressed the proliferation and migration of A549 cells in a dose-dependent manner. This effect may be attributed to cell cycle arrest at the G2/M phase and the induction of apoptosis, potentially via activation of the mitochondrial apoptotic pathway. Introducing a Schiff base and coordinating it with Cu(II) significantly enhanced the anticancer activity of carvacrol, with the copper–Schiff base complex showing the greatest potency by improving cellular uptake and strengthening interactions that promote cell-cycle arrest and apoptosis.

Alamri and colleagues developed a series of thymol and carvacrol derivatives to evaluate their anticancer potential. Among the synthesized compounds, the ethoxy-cyclohexyl analogues (**28** and **29,** Figure 3, Table 1), prepared through Williamson ether synthesis, consistently demonstrated the highest activity across a panel of ten cancer cell lines of diverse origins. The results were consistent with findings reported in the literature [57], where the cyclohexyl moiety has previously been shown to exhibit anticancer activity. Molecular docking results showed that compounds **28** and **29** fit deeply into the AKT1 protein’s binding pocket and form strong interactions with multiple amino acid residues. Overall, these findings suggest that thymol and carvacrol, if suitably modified could serve as potential anticancer agents with their activity likely linked to their ability to interact with the AKT1 protein [58].

Laamari et al. [59] synthesized a new series of thymol derivatives incorporating a pyrazole scaffold via 1,3-dipolar cycloaddition reactions between thymol-O-propargyl derivatives and diarylnitrilimines. The resulting compounds were screened for their cytotoxic potential against four human cancer cell lines: MCF-7 and MDA-MB-231 (breast adenocarcinoma), A-549 (lung adenocarcinoma), and HT-1080 (fibrosarcoma). Among the tested derivatives, compounds **30** and **31** (Figure 3, Table 1) demonstrated the most significant anticancer activity against A-549 cells, with IC_50_ values of 22.17 ± 1.34 µM and 23.79 ± 1.89 µM, respectively.

Blažíčková et al. [60] developed two novel thymol derivatives, including acetic acid thymol ester and thymol β-D-glucoside, with an emphasis on improving hydrophilicity. Compound **32** (Figure 3) was synthesized via BF_3_·Et_2_O-catalyzed glycosylation of thymol with 1,2,3,4,6-penta-O-acetyl-β-D-glucose in dichloromethane, followed by neutralization, extraction, chromatographic purification, and recrystallization to afford the product in 52% yield with 98.6% purity. Compound **33** (Figure 3, Table 1) was obtained by methanolysis (deacetylation) of 1-O-thymol-2,3,4,6-tetra-O-acetyl-β-D-glucoside using sodium methoxide in methanol under mildly basic conditions, followed by neutralization and solvent removal to afford the product in 83% yield. Their cytotoxic effect was evaluated against colorectal cancer cell lines (HT-29 and HCT-116) using the MTT assay. Remarkably, acetic acid thymol ester (**32**, Table 1) exhibited strong cyto/genotoxic activity at significantly lower concentrations (IC_50_~0.08 μM) compared to thymol (IC_50_~60 μM) after 24 h of treatment. In contrast, thymol β-D-glucoside (**33,** Table 1) displayed genotoxic effects only at much higher concentrations (~1000 μM). Both derivatives demonstrated dose- and time-dependent antiproliferative activity over 100 h, with no notable increase in micronuclei formation. Additionally, treated cells showed enhanced Reactive oxygen species (ROS) generation. These findings suggest that the anticancer potential of thymol derivatives is strongly influenced by their chemical structure. While further studies are required, thymol and its derivatives hold promising potential in the prevention and treatment of colorectal cancer, one of the most prevalent malignancies worldwide.

A study by Bsharat et al. [61] synthesized a novel thymol ester derivative and evaluated its antibacterial, anticancer, and antioxidant properties. Thymol was condensed with indole-3-carboxylic acid in dichloromethane at room temperature to yield compound 2-isopropyl-5-methylphenyl 1H-indole-3-carboxylate (**34**) (Figure 3, Table 1), which was structurally confirmed using FT-IR and NMR spectroscopy. Biological evaluation revealed that **34** exhibited notable cytotoxicity against various cancer cell lines while showing better protection of normal muscle cells compared to thymol. In antibacterial assays, **34** demonstrated enhanced activity against Gram-positive bacteria, particularly *S. aureus* and *S. epidermidis*, with inhibition zones surpassing even gentamicin against *S. epidermidis*. Furthermore, **34** displayed stronger antioxidant activity than thymol at lower concentrations. Collectively, these findings highlight **34** as a promising thymol-based derivative with improved biological potential.

Vasconcelos et al. [62] explored the anticancer potential of Morita–Baylis–Hillman (MBH) adducts synthesized from carvacrol. The synthesis involved reacting aromatic aldehydes with carvacrol acrylate as a Michael acceptor, producing stable adducts in 60–92% yields within 24 h. Among the twelve compounds evaluated using the MTT assay, compound **35** (Figure 3, Table 1), an acrylate/2-naphthyl adduct demonstrated the most potent anticancer activity. It exhibited significant cytotoxicity against SH-SY5Y neuroblastoma cells, with an IC_50_ of 8.7 µM after 72 h, making it 42 times more active than carvacrol (IC_50_ = 374.1 µM). Moreover, compound **35** displayed a selectivity index (SI) of 4.28, indicating strong selectivity toward cancer cells over normal cells. Mechanistic investigations revealed that its cytotoxic action was associated with caspase-3/7-mediated apoptosis in a concentration-dependent manner. In silico studies further indicated favorable pharmacokinetic properties, including efficient oral absorption. Toxicological evaluation through brine shrimp lethality and the Irwin test confirmed its low toxicity, underscoring its promise as a potential lead compound for anticancer drug development.

Başaran et al. [63] designed and synthesized two new series of benzenesulfonate-based thymol derivatives **36** and **37** (Figure 3, Table 1) with potential chemotherapeutic properties. These two compounds were obtained by refluxing equimolar amounts of 4-aminothymol and the corresponding benzenesulfonate derivative in ethanol, followed by cooling, filtration, drying, and recrystallization from ethanol. Their antiproliferative effects were evaluated against human lung adenocarcinoma (A549) and colorectal adenocarcinoma (DLD-1) cell lines using the MTT assay over 48 and 72 h. The compounds demonstrated notable cytotoxicity, with IC_50_ values ranging from 9.98 to 81.83 µM for A549 cells and 4.29 to 53.62 µM for DLD-1 cells, compared to cisplatin (6.65 µM and 9.91 µM, respectively). Among them, compound **36** showed the strongest activity, with IC_50_ values of 9.98 µM (A549) and 10.75 µM (DLD-1). Molecular docking studies revealed significant interactions of compounds **36** and **37** with cancer-related targets Bcl-2, VEGFR-2, EGFR, and HER2. The SAR findings reveal a clear relationship between the molecular structures and their cytotoxic properties. When compounds share identical substituents, a notable pattern emerges: attaching the benzene sulfonate group to the Schiff base at the para position generally increases cytotoxicity against the DLD-1 cell line but markedly reduces activity against A549 cells. In contrast, when the benzene sulfonate group is connected at the ortho position, compound **36**, which contains a chlorine substituent, consistently demonstrates strong cytotoxic effects in both cell lines, independent of incubation duration.

Sahin et al. [64] synthesized novel thymol-based Schiff bases and evaluated their anticancer, antimicrobial, and antioxidant activities. These compounds were synthesized via nitrosation of thymol to form 2-isopropyl-5-methyl-4-nitrosophenol, followed by its conversion to 4-amino-2-isopropyl-5-methylphenol through ammonolysis and reduction, after which the target compounds, **38** and **39** in Figure 3, Table 1 were obtained according to the reported literature procedure by Kumar et al., 2013 [65]. Cytotoxicity was tested against liver, colon, lung, and prostate cancer cell lines, revealing significant effects. Compound **38** showed strong activity against colon cancer cells (DLD-1, IC_50_ = 12.39 µM), while compound **39** was most effective against prostate cancer cells (PC3, IC_50_ = 7.67 µM), both outperforming cisplatin. Both compounds exhibited strong cytotoxic and antioxidant activities but only moderate antimicrobial effects. Molecular docking studies against EGFR, VEGFR-2, FAK, B-Raf, and PI3K confirmed favorable binding interactions, consistent with the experimental findings.

Akkoc et al. [66] synthesized and characterized a thymol-based molecule 2-isopropyl-5-methylphenol derivative **40** (Figure 3, Table 1) and evaluated its antiproliferative activity against three cancer cell lines and a healthy human cell line. Compound **40** was synthesized via nitrosation of 2-isopropyl-5-methylphenol with sodium nitrite in ethanol to afford 2-isopropyl-5-methyl-4-nitrosophenol, followed by conversion to 4-amino-2-isopropyl-5-methylphenol using thioacetamide in an NH_4_OH–H_2_O system, and subsequent condensation with 3,4,5-trimethoxybenzaldehyde in ethanol. The compound **40** exhibited notable cytotoxic effects on the tested cancer cells, as confirmed by microscopy. Molecular docking studies revealed its binding mode with the ABL1 target, while molecular dynamics simulations confirmed the stability of the resulting complexes. Density functional theory and computational pharmacokinetics analyses indicated that the compound’s antiproliferative activity is likely linked to ABL1 inhibition and that it possesses favorable drug-like properties. Overall, both experimental and computational results suggest that molecule **40** and its derivatives hold promise as potential anticancer agents.

Structural modification of carvacrol and thymol has used several key synthetic strategies, such as pharmacophore hybridization, heterocycle incorporation (e.g., triazoles, oxadiazoles), esterification/prodrug formation, metal complexation, and lipophilic bulky substitutions. Among these, hybridization with established anticancer pharmacophores and incorporation of heterocyclic linkers have consistently produced the most significant improvements in anticancer potency, often shifting activity to low micromolar or submicromolar ranges while introducing clearer target specificity (e.g., TS or kinase inhibition). Click-chemistry-derived triazole hybrids represent a particularly effective and versatile approach, balancing potency and, in some cases, selectivity. Esterification and lipophilic substitutions can markedly enhance cellular uptake and potency but show less consistent effects on cancer-cell selectivity, whereas metal complexation offers mechanistic engagement yet remains less extensively validated. Overall, strategies that combine rational hybridization with heterocycle incorporation appear most promising for improving both activity and selectivity.

**Table 1:** Anticancer activity of selected carvacrol/thymol-based derivatives against human cancer cell lines. IC_50_ values are reported as described in the original studies and should be interpreted within their respective experimental conditions.

Compounds	Evidence Level/Proposed Mechanism	Cell Lines Tested	Reference Drug/Comparator	Bibliography
**6**	G_2_/M arrest and caspase-dependent apoptosis in lung and breast cancer cells	IC_50_, µM: HT-1080 (7.10 ± 1.23); A-549 (14.40 ± 0.36); MCF-7 (19.64 ± 1.45); MDA-MB-231 (17.80 ± 2.74);	Doxorubicin: HT-1080 (6.21 ± 1.75); A-549 (7.41 ± 0.32); MCF-7 (6.28 ± 1.28); MDA-MB-231 (5.98 ± 1.54)	[44]
**7**	Broad cytotoxicity with limited selectivity vs. normal breast cells	IC_50_, µM: MDA-MB-231 (16.57 ± 1.14); MCF-7 (0.47 ± 1.14); MCF-12A (0.75 ± 1.30); DU145 (16.21 ± 1.08)	Carvacrol: (>200 across tested lines)	[45]
**8**–**10**	In silico: predicted inhibition of EGFR and PDGFR tyrosine kinases	NR	NR	[46]
**11**–**12**	In silico: predicted PIK3CA targeting via stable ligand–protein interactions.	NR	NR	[47]
**13**	TS inhibition causing G_2_ arrest and apoptosis	IC_50_, µM: MCF-7 (3.52 ± 0.53); HepG2 (1.02 ± 0.02); HCT-116 (4.12 ± 0.92)	Doxorubicin: MCF-7 (10.87 ± 0.73); HepG2 (6.63 ± 0.96); HCT-116 (6.96 ± 0.93)	[49]
**14**		IC_50_, µM: MCF-7 (4.21 ± 1.62); HepG2 (5.01 ± 0.71); HCT-116 (13.31 ± 2.38)		
**15**	In silico: predicted inhibition of EGFR, BRAF V600E, and tubulin	NR	NR	[52]
**16**	Moderate antiproliferative activity across colon and liver cancer cells	IC_50_, µM: HepG2 (51.3 ± 4.1); HCT116 (39.1 ± 5.8); SW480 (33.7 ± 6.6); SW620 (43.5 ± 7.5); HaCaT (>100)	Doxorubicin: HepG2 (0.38 ± 1.7); HCT116 (0.59 ± 0.02); SW480 (0.75 ± 0.1); SW620 (0.26 ± 0.1); HaCaT (0.29 ± 0.1)	[53]
**17**		IC_50_, µM: HepG2 (43.4 ± 3.1); HCT116 (28.6 ± 0.1); SW480 (33.7 ± 3.5); SW620 (61.8 ± 0.1); HaCaT (59.1 ± 3.1)		
**18**		IC_50_, µM: HepG2 (41.8 ± 2.5); HCT116 (30.5 ± 2.3); SW480 (29.5 ± 1.9); SW62 (49.6 ± 7.0); HaCaT (64.9 ± 5.1)		
**19**	TS inhibition; strongest TS-blocking effect within series	IC_50_, µM: MCF-7 (2.4); HCT-116 (3.1); HepG2 (1.8)	Doxorubicin: MCF-7 (1.2); HCT-116 (2.5); HepG2 (1.8). Fluorouracil: MCF-7 (18.74); HCT-116 (30.68); HepG2 (28.65).	[48]
**20**		IC_50_, µM: MCF-7 (1.1); HCT-116 (2.6); HepG2 (1.4)		
**21**		IC_50_, µM: MCF-7 (1.3); HCT-116 (3.8); HepG2 (2.5)		
**22**	Predicted EGFR and BRAF inhibition with selective cytotoxicity.	IC_50_, µM: A549 (9.953 ± 0.01); BEAS-2B (36.65 ± 0.55)	Doxorubicin: A549 (11.54 ± 0.01); BEAS-2B (93.8 ± 1.41)	[54]
**23**		IC_50_, µM: A549 (9.24 ± 0.01); BEAS-2B (39.15 ± 0.59)		
**24**		IC_50_, µM: A549 (10.5 ± 0.01); BEAS-2B (41.42 ± 0.62)		
**25**–**26**	Predicted to inhibit EGFR through stable and favorable protein–ligand interactions.	NR	NR	[55]
**27**	Induces G2/M arrest and mitochondrial apoptosis in A549 cells	IC_50_, µg/mL: A549 (233.39 ± 4.18)	NR	[56]
**28**–**29**	In silico docking: predicted AKT1 pocket binding (no experimental cytotoxicity data).	NR	NR	[58]
**30**	NR	IC_50_, µM: HT-1080 (26.41 ± 1.39); A-549 (22.17 ± 1.34); MCF-7 (32.08 ± 0.81); MDA-MB-231 (6.34 ± 0.56)	Doxorubicin: HT-1080 (26.41 ± 1.39); A-549 (5.39 ± 0.39); MCF-7 (5.25 ± 1.73); MDA-MB-231(6.77 ± 0.12)	[59]
**31**		IC_50_, µM: HT-1080 (25.65 ± 0.84); A-549 (23.79 ± 1.89); MCF-7 (28.24 ± 2.13); MDA-MB-231 (34.81 ± 0.66)		
**32**	ROS-mediated cytotoxicity and DNA damage (structure-dependent effects	IC_50_, µg/mL: HT-29 (5.8 × 10^4^ ± 7748.5); HCT-116 (6.2 × 10^4^ ± 7018.2)	Thymol: HT-29 (8.7 × 10^3^ ± 1039.4); HCT-116 (3.0 × 10^4^ ± 7231.1)	[60]
**33**		IC_50_, µg/mL: HT-29 (5.3 × 104 ± 5360.1); HCT-116 (7.9 × 104 ± 3002.5)		
**34**	Selective cytotoxic and cytostatic effects with enhanced antioxidant activity	IC_50_, µg/mL: Hela cytotoxic (14.50); Hela cytostatic (0.094); L6 cytotoxic (8.066); L6 cytostatic (7.26); MCF7 cytotoxic (17.21); MCF7 cytostatic (7.08); PC3 cytotoxic (19.98); PC3 cytostatic (10.26); HepG2 cytotoxic (17.13); HepG2 cytostatic (13.04)	Thymol: Hela cytotoxic (13.90); Hela cytostatic (0.18); L6 cytotoxic (9.27); several values NR	[61]
**35**	Caspase-3/7-mediated apoptosis; selective toward neuroblastoma cells.	IC_50_, µM: SH-SY5Y (23.36 ± 1.50); HEK293 (>100)	Carvacrol: SH-SY5Y (374.1 ± 1.18); HEK293 (350.1 ±1.04)	[62]
**36**	Multi-target interactions (Bcl-2, VEGFR-2, EGFR, HER2); inhibits proliferation	IC_50_, µM: A549 (9.98); DLD-1 (10.75)	Cisplatin: A549 (6.65); DLD-1 (9.21)	[63]
**37**		IC_50_, µM: A549 (46.85); DLD-1 (4.29)		
**38**	Antiproliferative effects across liver, colon, lung, and prostate cancer cells	IC_50_, µM: HepG2 (67.83); DLD-1 (12.39); A549 (73.93); PC3 (35.65)	Cisplatin: HepG2 (69.36); DLD-1 (19.16); A549 (11.58); PC3 (16.27)	[64]
**39**		IC_50_, µM: HepG2 (74.41); DLD-1 (16.76); A549 (56.01); PC3 (7.67);		
**40**	Likely ABL1 kinase inhibition producing antiproliferative effects.	IC_50_, µM: MDA-MB-231(75.13); DLD-1 (62.07); HepG2 (71.49); Wl-38 (117.90)	Cisplatin: MDA-MB-231 (11.94); DLD-1 (NT); HepG2 (57.38); Wl-38 (50.59)	[66]

NR = not reported. IC_50_ values were extracted from independent studies conducted under heterogeneous experimental conditions (e.g., assay type, incubation time, cell density, and calculation methods). Therefore, these values should be interpreted within each study’s context and are presented to illustrate activity trends rather than to provide direct cross-study potency rankings.

### 4.2. Menthol

Menthol is a naturally occurring monocyclic monoterpene alcohol predominantly found in the EOs of *Mentha* species [67] and is widely used in pharmaceutical, cosmetic, and food industries due to its favorable safety profile and bioactivity [68,69,70]. In recent years, menthol has gained significant attention in cancer research as both a bioactive compound and a versatile scaffold for chemical modification. Extensive evidence from in vitro and in vivo studies [71,72], supported by limited clinical observations, indicates that menthol exhibits inhibitory and therapeutic effects against several cancer types, including liver [73], skin [74], and prostate cancers [75]. The anticancer activity of menthol is attributed to multiple mechanisms, such as the suppression of cancer cell proliferation [76] and metastasis [24], inhibition of tumor angiogenesis [77], and induction of apoptosis [78]. Structural derivatization of menthol has been shown to overcome its moderate intrinsic potency, leading to enhanced anticancer activity, improved selectivity, and diversified mechanisms of action. Consequently, menthol-based derivatives have emerged as promising candidates in anticancer drug discovery, warranting systematic evaluation of their SARs and underlying molecular mechanisms.

**Figure 4:**
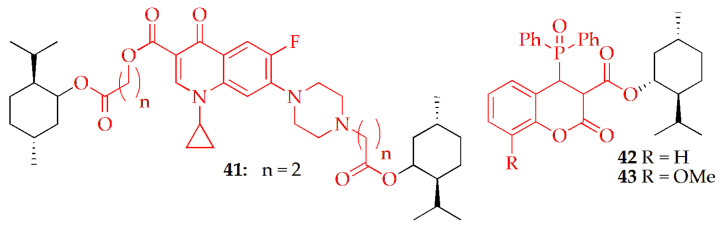
Menthol-based derivatives synthesized via hybridization strategies (**41**–**43**).

Szostek et al. [53] synthesized a series of ciprofloxacin-based menthol derivatives and evaluated their anticancer potential. Menthol-based ciprofloxacin hybrid derivatives were synthesized by reacting ciprofloxacin with the corresponding menthol ester in DMF in the presence of NaHCO_3_ at 70 °C, followed by acidic work-up, extraction, and purification by silica gel column chromatography. Among them, compound **41** (Figure 4, Table 2) exhibited strong cytotoxic activity against cancer cells while remaining non-toxic to normal cells. Their selectivity index (SI) values ranged from 1.9 to 3.4, notably higher than that of doxorubicin (0.14–1.11), indicating better cancer selectivity. Molecular docking studies revealed that these active derivatives effectively bound to topoisomerase II (DNA gyrase) in complex with DNA (PDB ID: 5BTC). The analysis identified compound **41** as the leading molecule. Furthermore, it was noted that modifications disrupting the 3-oxo-4-carboxylic acid core of the ciprofloxacin scaffold reduced antibacterial and anticancer activity, confirming this moiety as the key site for DNA-gyrase interaction.

Szwaczko et al. [79] synthesized menthol-modified coumarin esters and 3-phosphorylated coumarins through a Michael addition of P(O)H groups to the coumarin scaffold, yielding 3,4-dihydrocoumarin derivatives. The reaction achieved excellent yields (89–98%) under mild conditions and using eco-friendly solvents such as acetonitrile or water. The obtained compounds were evaluated for cytotoxicity against human cancer cell lines colorectal (SW480, SW620), prostate (PC3), and breast (MDA-MB-231), as well as normal keratinocytes (HaCaT) using the MTT assay, with doxorubicin and cisplatin as standards. Among them, compounds **42** and **43** (Figure 4, Table 2) exhibited the most potent activity and were further examined for apoptosis induction, IL-6 inhibition, and antiproliferative properties. Despite **42** and **43** being tested as diastereomeric mixtures due to separation limitations, they demonstrated notable cytotoxicity and selectivity toward specific cancer cell lines.

Menthol-based anticancer derivatives have primarily relied on two principal modification strategies, such as pharmacophore hybridization and scaffold conjugation, each influencing potency and selectivity in distinct ways. Hybridization of menthol with established anticancer pharmacophores, exemplified by ciprofloxacin–menthol conjugates, appears to be the most effective strategy for enhancing both cytotoxic potency and cancer selectivity, likely because the menthol moiety increases lipophilicity and membrane permeability [24] while the parent drug core preserves target engagement (e.g., topoisomerase II/DNA gyrase binding) [80]. This approach yielded derivatives with improved selectivity indices compared with standard drugs, highlighting the advantage of retaining a validated pharmacophoric core while modulating physicochemical properties. In contrast, scaffold conjugation strategies such as menthol-modified coumarin esters and phosphorylated coumarins produced compounds with moderate-to-strong antiproliferative activity and apoptosis-related effects, but their selectivity was more variable and sometimes limited by testing as diastereomeric mixtures, which complicates precise SAR interpretation. Overall, pharmacophore hybridization with clinically relevant scaffolds appears most effective in improving both anticancer activity and selectivity, whereas simpler esterification or scaffold-decoration approaches mainly enhance cytotoxicity but provide less consistent selectivity gains; however, conclusions remain tentative given the limited number of comparable compound series and the predominance of in vitro evaluations without extensive in vivo or pharmacokinetic validation. 

**Table 2:** Anticancer activity of selected menthol-based hybrid compounds against human cancer cell lines. IC_50_ values are reported as provided in the original studies and should be interpreted within their respective experimental contexts rather than as directly comparable metrics across studies.

Compounds	Proposed Mechanism	Cell Lines (IC_50_ µM)	Reference Drug/Comparator	Bibliography
**41**	Inhibition of topoisomerase II/DNA complex affecting replication	HepG2 (36.8 ± 3.8); HCT116 (27.1 ± 3.1); SW480 (30.3 ± 1.2); SW620 (38.6 ± 3.8); HaCaT (45.5 ± 5.1);	Doxorubicin: HepG2 (0.38 ± 1.7); HCT116 (0.59 ± 0.02; SW480 (0.75 ± 0.1); SW620 (0.26 ± 0.1); HaCaT (0.29 ± 0.1)	[53]
**42**	Induction of apoptosis and suppression of IL-6 signaling in colorectal, prostate, and breast cancer cells	SW480 (4.6 ± 1.01); SW620 (6.8 ± 0.55); PC3 (9.8 ± 2.02); MDA-MB-231 (25.3 ± 4.59); HaCaT (15 ± 2.76);	Cisplatin: SW480 (10.4 ± 0.90; SW620 (6.7 ± 1.10); PC3 (13.2 ± 2.10); MDA-MB-231 (7.3 ± 0.80); HaCaT (6.3 ± 0.70)	[79]
**43**		SW480 (37.4 ± 2.45); SW620 (19.8 ± 0.0.63); PC3 (9.9 ± 1.42); MDA-MB-231 (47.6 ± 4.73); HaCaT (27.6 ± 3.12)		

IC_50_ values were extracted from independent studies conducted under heterogeneous experimental conditions (e.g., assay type, incubation time, cell density, and calculation methods). Therefore, these values should be interpreted within each study’s context and are presented to illustrate activity trends rather than to provide direct cross-study potency rankings.

### 4.3. Eugenol

Eugenol is a naturally occurring phenolic monoterpene widely distributed in essential oils, particularly from *Syzygium aromaticum* (cloves), and is known for its diverse pharmacological properties [81,82]. Increasing evidence has highlighted eugenol as a promising anticancer scaffold due to its ability to interfere with cancer cell proliferation, induce apoptosis, and modulate oxidative stress and inflammatory pathways [83]. Nevertheless, the therapeutic application of eugenol is constrained by moderate potency and limited selectivity [84]. Consequently, extensive structural modifications such as the development of Mannich bases, chalcone–triazole hybrids, β-amino alcohols, isothiocyanates, ester derivatives, and metal-linked conjugates have been explored to enhance its anticancer efficacy and pharmacokinetic profile. This section reviews recent advances in the synthesis, biological evaluation, and mechanistic insights of eugenol-derived compounds, emphasizing their potential role in anticancer drug discovery.

**Figure 5:**
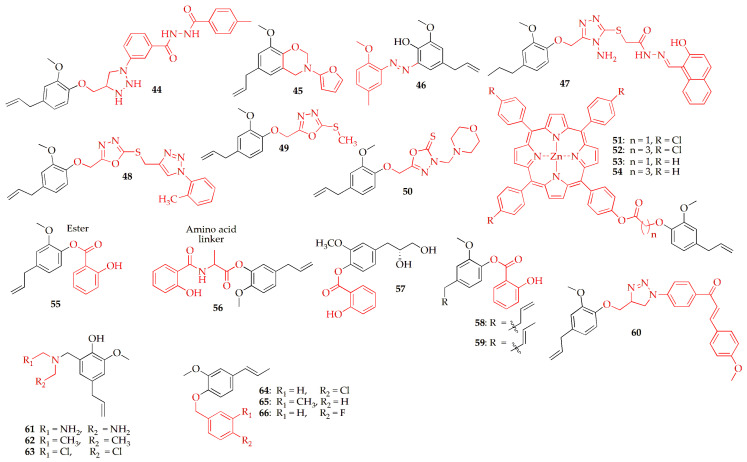
Chemical structures of eugenol-based derivatives synthesized via hybridization strategies.

Alam et al. [85] synthesized and characterized novel eugenol-based 1,2,3-triazole derivatives using NMR, MS, IR, and elemental analysis, and evaluated their anticancer activity against breast cancer cell lines. These derivatives were synthesized via O-propargylation of eugenol followed by CuAAC click coupling with 3-azidobenzoic acid to form a triazole intermediate, which was subsequently derivatized through ethyl chloroformate activation and amine or hydrazide substitution in the presence of TEA to afford the target products. Among them, compound **44** (Figure 5, Table 3) having a tolyl group, exhibited the strongest cytotoxicity, surpassing eugenol and showing IC_50_ values of 6.91 μM (MDA-MB-231) and 3.15 μM (MCF-7), comparable to doxorubicin. Further analysis revealed that compound **44** induced cell cycle arrest at the S and G_2_ phases in MCF-7 cells, highlighting eugenol-triazole conjugates as promising anticancer leads.

Rudyanto and co-workers investigated the in vivo anticancer effects of benzoxazine and aminomethyl derivatives of eugenol in a mouse fibrosarcoma model induced by benzo(a)pyrene. The compounds were administered orally at doses of 20, 40, and 80 mg/kg body weight daily for 30 days. All tested derivatives significantly reduced cancer incidence and tumor weight, with benzoxazine derivatives showing slightly higher activity than aminomethyl ones. The most potent compound was Compound **45** (Figure 5, Table 3), 6-allyl-3-(furan-2-ylmethyl)-8-methoxy-3,4-dihydro-2H-benzo(e)(1,3)oxazine. Overall, all four eugenol-based compounds demonstrated notable anticancer activity in this fibrosarcoma model [86].

Dandge et al. [87] synthesized a series of six aryl-azo-eugenol derivatives via the conventional diazotization–coupling method, where diazonium salts were coupled with eugenol. The structures of the synthesized compounds were confirmed using ^1^H NMR, ^13^C NMR, FT-IR, and mass spectrometry. Among them, compound **46** (Figure 5, Table 3) demonstrated strong cytotoxic activity against the MDA-MB-231 human breast cancer cell line, exhibiting potency comparable to Adriamycin (10 μg/mL). Overall, **46** was identified as the most potent compound, and its enhanced activity was attributed to the presence of methoxy groups on both aromatic rings, which may contribute to its chemotherapeutic mechanism.

Alam et al. [88] synthesized a series of eugenol-based 1,2,4-triazole derivatives to explore their anti-COX-2 and antiproliferative potential. Structural characterization of the new compounds was confirmed using advanced spectroscopic techniques. Among the derivatives, compound **47** (Figure 5, Table 3) exhibited remarkable activity, showing cytotoxic potency comparable to doxorubicin against MDA-MB-231 and PC-3 cancer cell lines, with IC_50_ values of 1.42 and 5.69 μM, respectively. It also demonstrated strong COX-2 inhibition (IC_50_ = 0.28 μM). In silico studies revealed that compound 46 is non-carcinogenic, non-mutagenic, and possesses favorable drug-likeness and pharmacokinetic properties. Molecular docking further supported its high affinity for COX-2, aligning with the biological results. Overall, compound **47** emerged as a promising dual COX-2 inhibitor and antiproliferative agent with potential application in cancer therapy [89]. The same Authors synthetically modified eugenol to yield a series of 1,3,4-oxadiazole derivatives designed as TS inhibitors. Among the series, compounds **48** and **49** (Figure 5, Table 3) exhibited the most potent antiproliferative effects, with IC_50_ values of 0.99 and 1.25 μM (MCF-7) and 1.17 and 0.26 μM (PC3), surpassing doxorubicin in efficacy. Both compounds were strong TS inhibitors, showing IC_50_ values of 0.61 μM and 0.56 μM, outperforming pemetrexed (IC_50_ = 2.81 μM). They also induced S-phase cell cycle arrest and apoptosis in cancer cells, similar to 5-fluorouracil. ADMET analysis indicated favorable pharmacokinetic profiles, high oral absorption (96.6% for compound **48** and 95% for compound **49**), and absence of carcinogenicity or toxicity. Molecular docking studies revealed binding patterns analogous to 5-fluorouracil within the TS active site. Overall, compounds **48** and **49** demonstrated potent TS inhibition and strong anticancer potential, positioning them as promising candidates for chemotherapeutic development.

Nazreen et al. [90] synthesized a series of eugenol-derived 1,3,4-oxadiazole molecular hybrids incorporating N-substituted acetamide and Mannich base structures for anticancer evaluation. These hybrid molecules were synthesized from eugenol via sequential O-alkylation, hydrazide formation, and cyclization with carbon disulfide to afford a key intermediate, which upon Mannich-type reaction with formaldehyde and aliphatic amines in ethanol yielded the final product. Among them, compound **50** (Figure 5, Table 3), containing a morpholine moiety, displayed the strongest cytotoxicity with IC_50_ values of 1.71 μM (MCF-7), 1.84 μM (SKOV3), and 1.1 μM (PC-3). It also effectively inhibited TS with an IC_50_ of 0.81 μM. Cellular studies revealed that compound **50** induces apoptosis and causes S-phase cell cycle arrest in PC-3 cancer cells. Molecular docking suggested that it acts as a transition-state inhibitor, showing similar binding interactions to 5-fluorouracil. Furthermore, in silico pharmacokinetic and DFT analyses confirmed its potential for good oral bioavailability. Overall, compound **50** emerged as a promising TS inhibitor capable of disrupting DNA synthesis and mitigating DNA damage in prostate cancer cells [91].

Li et al. [92] successfully prepared a series of porphyrin–butylphenol derivatives, all of which demonstrated strong singlet oxygen–generating capacity. These derivatives were synthesized by O-alkylation and hydrolysis of eugenol to afford carboxylic acid derivatives, followed by porphyrin formation, acyl chloride activation, coupling with porphyrins, and final zinc metalation to yield the corresponding zinc–porphyrin–eugenol hybrids. Their in vitro anticancer evaluation revealed that compounds **51** and **52** (Figure 5, Table 3) exhibited notable inhibition against HepG2 cells, while **51**, **53**, **54** (Figure 5, Table 3) showed more potent activity against A549 cells than the raw eugenol. Under light irradiation, compound **51** achieved an IC_50_ of 66.06 μM in HepG2 cells, and compound **53** recorded an IC_50_ of 66.52 μM in A549 cells, both outperforming the reference drug 5-fluorouracil. Additionally, the zinc-chelated derivatives demonstrated superior antitumor activity compared to their free-base porphyrin counterparts.

Warsito et al. [93] explored a hybrid design strategy for developing potential anticancer agents by combining two essential oil-derived molecules such as salicylic acid and eugenol with or without an amino acid (alanine) linker. The eugenol-based salicylic acid hybrid with an amino linker was synthesized via a two-step DCC-mediated method involving initial amidation of salicylic acid with alanine, followed by esterification of the resulting amino acid intermediate with eugenol in the presence of DCC and DMAP, yielding the final conjugate after standard work-up. Eugenyl salicylate without the linker was synthesized via DCC/DMAP-mediated esterification of salicylic acid with eugenol under solvent-free conditions, followed by aqueous work-up, drying, and solvent removal to afford the ester-linked hybrid compound. The study integrated computational and synthetic approaches, utilizing ADMET analysis, Lipinski’s rule evaluation, and molecular docking against seven cancer-related receptors (MMP9, MMP2, CDK2, P53, BAK, EGFR, and MRPR). Nonlinker hybrid (**55**) (Figure 5, Table 3) was synthesized via esterification reactions catalyzed by DCC and DMAP, while alanine-linked hybrid (**55**–**56**) was obtained through a two-step amidation and esterification process. The synthesized compounds were characterized using TLC, FTIR, and LC-MS. ADMET and Lipinski analyses confirmed that both types of hybrids possess favorable drug-like properties. Docking studies revealed that nonlinker hybrids exhibited strong affinity for the BAK receptor (PDB ID: 6UXM), suggesting pro-apoptotic potential, whereas alanine-linked hybrids showed activity against both the MMP9 enzyme (PDB ID: 4H1Q), associated with cancer metastasis, and the BAK receptor. The synthesis yielded 59.85% for nonlinker hybrids and 93.89% for alanine-linked hybrids, indicating successful and efficient synthesis of promising anticancer candidates.

Fadilah et al. [94] synthesized a series of new eugenyl benzoate derivatives (2-methoxy-4-(prop-2-en-1-yl)phenyl benzoates) aimed at inhibiting HT29 colorectal cancer cells. The compounds were prepared through a sequence of reactions, including esterification, demethylation, halohydrin formation, and the Sharpless reaction. Cytotoxicity assays were conducted to assess their activity against HT29 cells, while QSAR analysis was used to correlate structural features with biological activity. Ten novel compounds were successfully synthesized, with IC_50_ values ranging from 26.56 to 286.81 μmol/mL. Among them, compound **57** (4-[(2S)-2,3-dihydroxypropyl]-2-methoxyphenyl 2-hydroxybenzoate) (Figure 5, Table 3) exhibited the strongest inhibitory effect, acting as a potent BCL-2 inhibitor, outperforming both eugenol and the other synthesized derivatives. QSAR analysis yielded the regression equation: 1/IC_50_ = −0.865 − 0.210(LogP)^2^ + 1.264(LogP) − 0.994(CMR) (n = 10; r = 0.706; SE = 0.21; F = 0.497; sig = 7.86), indicating that both hydrophobicity (LogP) and steric factors (CMR) influence cytotoxicity, with LogP having a more significant impact. Overall, compound **57** emerged as the most active BCL-2 inhibitor, and QSAR results suggest that hydrophobic interactions play a key role in enhancing the colorectal anticancer activity of these eugenol-based derivatives.

Pangesti et al. [95] designed and evaluated eugenol ester derivatives through molecular docking studies against five breast cancer-related proteins: MMP9, MMP2, Cyclin A2 (CCNA2), BAK, and P53. The most promising compounds identified from the in silico screening were synthesized via Steglich esterification using DCC and DMAP as catalysts. Among the tested derivatives, eugenyl salicylate (**58**) and isoeugenyl salicylate (**59**) (Figure 5, Table 3) exhibited the strongest binding affinities toward the MMP9 protein, with binding energies of −8.5 and −8.9 kcal/mol, respectively, indicating their potential as effective anticancer agents.

Buduma et al. [96] synthesized and evaluated novel Mannich base and triazole–chalcone derivatives of eugenol for their biological activities. Mannich base derivatives were synthesized by condensation of eugenol with formaldehyde and substituted secondary amines, while triazole chalcone derivatives were prepared via O-propargylation of eugenol followed by Cu(I)-catalyzed 1,3-dipolar cycloaddition with various azides to afford 1,2,3-triazolyl-eugenol derivatives. Among the tested compounds, the 4-methoxy chalcone triazole derivative **60** (Figure 5) (IC_50_ = 33.05 μM) and the di-amine Mannich derivative **61** (Figure 5) (IC_50_ = 32.92 μM) exhibited strong antiproliferative effects against HepG2 liver cancer cells, surpassing doxorubicin (IC_50_ = 37.29 μM). Several compounds, including **62** (17.75 μM), **61** (17.02 μM), and **63** (Figure 5) (20.12 μM), also demonstrated moderate to high cytotoxicity toward MCF-7 breast cancer cells as shown in Table 3.

Nafie et al. [97] synthesized a series of semi-synthetic isoeugenol derivatives and evaluated their potential as safe and effective anticancer agents. The compounds were synthesized via base-catalyzed O-alkylation of isoeugenol with substituted benzyl chlorides in ethanol under reflux, followed by aqueous work-up and purification by silica gel column chromatography. The compounds were tested for cytotoxicity against MCF-7 breast cancer cells and their selectivity assessed using the normal MCF-10A cell line. Among the derivatives, compounds **64**, **65**, and **66** (Figure 5, Table 3) exhibited superior activity compared to the standard drug 5-fluorouracil, with IC_50_ values of 6.59, 8.07, and 9.63 μM, respectively, while maintaining selectivity toward cancer cells. Further studies on compound **64** revealed that it inhibited MCF-7 colony formation by 87.5% and reduced ERα concentration to 395.7 pg/mL compared to 1129 pg/mL in untreated cells. It also significantly induced apoptosis, increasing total apoptotic cell death by 9.16-fold (18.7%) and causing cell cycle arrest at the G2/M phase. Molecular investigations confirmed upregulation of pro-apoptotic and downregulation of anti-apoptotic genes and proteins, alongside enhanced antioxidant enzyme activities (GSH, CAT, SOD). In vivo studies further validated the efficacy of compound **64**, showing 47.6% inhibition of tumor growth versus 22.9% for 5-fluorouracil, while restoring hematological, biochemical, and histopathological parameters to near-normal levels. Overall, compound **64** emerged as a promising isoeugenol-derived anticancer agent with notable selectivity toward breast cancer cells, acting through apoptosis induction and ERα downregulation.

**Figure 6:**
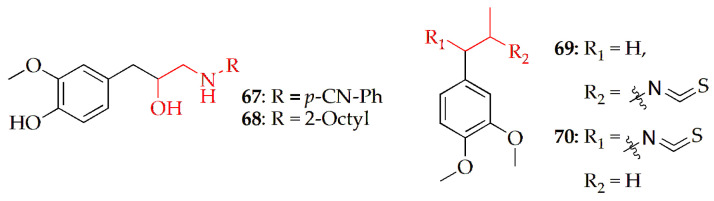
Chemical structures of eugenol-based-β-amino alcohol derivatives (**67**–**68**) and isothiocyanate derivatives (**69**–**70**).

Teixeira et al. [98] evaluated the cytotoxic potential of several β-amino alcohol derivatives of eugenol against AGS (gastric cancer) and A549 (lung cancer) cell lines. Eugenol-based β-amino alcohol derivatives were prepared via epoxidation of eugenol with m-chloroperbenzoic acid followed by nucleophilic ring opening of the resulting epoxide with various aromatic and aliphatic amines in ethanol–water. Their findings indicated that compounds **67** and **68** (Figure 6, Table 3) exhibited the strongest cytotoxic effects at 100 µM, showing greater activity than eugenol itself. Additionally, compound **68** notably enhanced caspase-3 activity, an enzyme associated with the induction of apoptosis. These results suggest that compound **68** may serve as a promising anticancer candidate and a lead molecule for the development of more potent anticancer agents.

Kazal et al. [99] synthesized two novel isothiocyanate derivatives of eugenol-methyl eugenol isothiocyanate (**69**) and methyl isoeugenol isothiocyanate (**70**) (Figure 6, Table 3). Compound **68** was prepared by reacting methyl eugenol with in situ–generated thiocyanic acid (from KSCN and KHSO_4_) in chloroform at room temperature for 24 h, followed by solvent removal, drying under a nitrogen stream, and purification via silica gel column chromatography. Compound **70** was synthesized by reacting methyl isoeugenol with in situ–generated thiocyanic acid (from KSCN and KHSO_4_) in chloroform at room temperature, followed by neutralization, solvent evaporation, and purification by silica gel column chromatography. The anticancer potential of both compounds was evaluated using in vitro MTT assays against 4T1 breast cancer cells and normal Vero cells, along with in silico molecular docking (Pyrx 9.0) against the MMP9 enzyme (PDB ID: 4H1Q). The results showed that (**69**) exhibited stronger cytotoxicity (IC_50_ = 21.08 μM) compared to (**70**) (IC_50_ = 258.69 μM) against 4T1 cells. Both compounds demonstrated moderate toxicity toward Vero cells, with IC_50_ values of 160.59 μM (**69**) and 92.16 μM (**70**). The selectivity index (SI) values were 0.26 for **69** and 4.37 for **70**, indicating that **70** is selectively toxic to cancer cells (SI > 3). Molecular docking analysis revealed that both **69** and **70** had comparable binding affinities (−8.2 kcal/mol) to standard anticancer drugs, supporting their potential as effective anticancer candidates. Overall, the study concluded that **70**, in particular, shows promising in vitro and in silico anticancer activity and could serve as a lead compound for future anticancer drug development.

Across eugenol derivatives, several structural modification strategies have been explored, including heterocycle incorporation (triazoles, oxadiazoles), pharmacophore hybridization, esterification/amide formation, metal–porphyrin conjugation, Mannich base formation, and introduction of bioactive functional groups such as isothiocyanates or β-amino alcohols. Among these, heterocycle-based hybridization, particularly 1,2,3-triazole and 1,3,4-oxadiazole conjugation emerges as the most consistently effective approach for enhancing anticancer potency, frequently yielding low-micromolar or submicromolar IC_50_ values alongside defined mechanisms such as thymidylate synthase or COX-2 inhibition and cell cycle arrest (Table 3). Pharmacophore-linked hybrids (e.g., salicylic acid or porphyrin conjugates) broaden mechanistic scope, enabling apoptosis induction or photodynamic ROS-mediated cytotoxicity, but show more variable selectivity and often rely on in silico validation. Mannich base and chalcone–triazole derivatives provide moderate improvements in cytotoxicity, suggesting that simple functionalization can enhance activity but with less predictable target specificity. In contrast, isoeugenol derivatives and certain ester or isothiocyanate modifications demonstrate notable selectivity toward cancer cells, indicating that tuning lipophilicity and electronic properties can improve therapeutic windows even when potency gains are modest. Overall, heterocycle-driven hybridization combined with rational pharmacophore integration appears most effective in improving both anticancer activity and mechanistic relevance, whereas simpler esterification or substitution strategies primarily modulate potency or selectivity in a scaffold-dependent manner; however, the predominance of in vitro assays and occasional reliance on computational predictions underscores the need for cautious interpretation and further in vivo and pharmacokinetic validation.

**Table 3:** Anticancer activity of selected eugenol-based derivatives against human cancer cell lines. IC_50_ values are reported as described in the original studies and should be interpreted within their respective experimental conditions rather than as directly comparable metrics across studies.

Compounds	Evidence Level/Proposed Mechanism	Cell Lines Tested	Reference Drug/Comparator	Bibliography
**44**	Induces S- and G_2_-phase arrest in breast cancer cells.	IC_50_, μM: MDA-MB-231 (6.91); MCF-7 (3.15)	Doxorubicin: MDA-MB-231 (6.58); MCF-7 (3.21) Eugenol: MDA-MB-231 (41.14); MCF-7 (38.7)	[85]
**45**	In vivo: tumor suppression in benzo(a)pyrene-induced mouse fibrosarcoma model; apoptosis implicated	NR	NR	[86]
**46**	NR	GI_50_, μM: MDA-MB-231 (10)	Adriamycin: MDA-MB-231 (10)	[87]
**47**	COX-2 inhibition with docking-supported binding to active site	IC_50_, μM: MDA-MB-231 (1.42 ± 0.68); HCT-116 (3.41 ± 0.52); PC-3 (5.69 ± 0.43)	Doxorubicin: MDA-MB-231 (1.39 ± 0.11); HCT-116 (2.36 ± 0.04); PC-3 (5.51 ± 0.70)	[89]
**48**	TS inhibition causing S-phase arrest and apoptosis	IC_50_, μM: MCF-7(0.99 ± 0.33); SKOV3 (2.61 ± 0.68); PC3 (1.17 ± 0.13)	Doxorubicin: MCF-7 (1.74 ± 0.34); SKOV3 (2.88 ± 0.68); PC3 (2.61 ± 0.23)	[88]
**49**		IC_50_, μM: MCF-7 (1.25 ± 0.26); SKOV3 (1.51 ± 0.41); PC3 (0.26 ± 0.08)		
**50**	Induce apoptosis and arrest the cell cycle at the S phase in PC-3 carcinoma. TS transition-state inhibition (mechanistic inference)	IC_50_, μM: MCF-7 (1.71 ± 0.95); SKOV3 (1.84 ± 0.27); PC-3 (1.1 ± 0.07)	Doxorubicin: MCF-7 (1.74 ± 0.34); SKOV3 (2.88 ± 0.68); PC-3 (2.61 ± 0.23)	[90,91]
**51**	Photodynamic ROS/singlet oxygen–mediated apoptosis	IC_50_, μM: HepG2 (69.25 ± 2.67); A549 (73.17 ± 2.20)	5-Fluorouracil: HepG2 (83.44 ± 4.53); A549 (80.86 ± 2.40)	[92]
**52**		IC_50_, μM: HepG2 (66.06 ± 2.29); A549 (79.96 ± 2.61)		
**53**		IC_50_, μM: HepG2 (80.44 ± 3.16); A549 (66.52 ± 2.69)		
**54**		IC_50_, μM: HepG2 (84.18 ± 1.76); A549 (78.62 ± 2.66)		
**55–56**	In silico: predicted pro-apoptotic (BAK) and anti-metastatic (MMP9) activity	NR	NR	[93]
**57**	BCL-2 inhibition promoting apoptosis; stronger activity than parent eugenol	IC_50_, µmol/mL: HT29 (26.56 ± 0.52)	Doxorubicin: HT29 (6.11 ± 0.76) Eugenol: HT29 (172.41 ± 1.14)	[94]
**58** and **59**	In silico: predicted MMP-9 inhibition (metastasis-related pathways).	NR	NR	[95]
**60**	Suppresses proliferation in liver and breast cancer cells	IC_50_,μM: A549 (>100); HepG2 (33.05); MCF-7 (32.77); SKOV3 (>100)	Doxorubicin: A549 (07.22); HepG2 (37.29); MCF-7 (02.96); SKOV3 (11.34)	[96]
**61**		IC_50_,μM: A549 (16.43); HepG2 (32.92); MCF-7 (17.02); SKOV3 (19.06)		
**62**		IC_50_,μM: A549 (>100); HepG2 (77.72); MCF-7 (17.75); SKOV3 (21.78)		
**63**		IC_50_,μM: A549 (>100); HepG2 (>100); MCF-7 (20.12); SKOV3 (>100)		
**64**	ERα downregulation, apoptosis induction, and G_2_/M arrest; in vitro and in vivo breast tumor inhibition	IC_50_,μM: MCF-7 (6.59 ± 0.4); MCF-10A (28.0 ± 0.7)	5-Fluorouracil: MCF-7 (30.93 ± 1.8); MCF-10A (20.4 ± 0.5)	[97]
**65**		IC_50_,μM: MCF-7 (8.07 ± 0.5); MCF-10A (18.1 ± 0.5)		
**66**		IC_50_,μM: MCF-7 (9.63 ± 0.6); MCF-10A (17.3 ± 0.5)		
**67**–**68**	Caspase-3-associated apoptosis in AGS and A549 cells (qualitative evidence)	NR	NR	[98]
**69**	Selective cytotoxicity in breast cancer model; MMP-9 inhibition suggested by docking	IC_50_, μg/mL: 4T1 Cell (258.69); Vero Cell (160.59)	NR	[99]
**70**		IC_50_,μg/mL: 4T1 Cell (21.08); Vero Cell (92.16)		

NR = not reported. IC_50_ values were extracted from independent studies conducted under heterogeneous experimental conditions (e.g., assay type, incubation time, cell density, and calculation methods). Therefore, these values should be interpreted within each study’s context and are presented to illustrate activity trends rather than to provide direct cross-study potency rankings.

### 4.4. Cinnamaldehyde

Cinnamaldehyde is a naturally occurring phenylpropanoid predominantly found in *Cinnamomum* species and is well recognized for its broad spectrum of biological activities [100,101]. In recent years, cinnamaldehyde has attracted considerable interest as a promising anticancer agent due to its ability to modulate multiple cancer-related signaling pathways [102,103]. However, its clinical applicability is limited by moderate potency, instability, and rapid metabolism [104,105,106]. To address these challenges, extensive structural modification of cinnamaldehyde has been pursued, leading to the development of diverse derivatives including chalcones, hydrazones, Schiff bases, and polymeric or nanoparticle-based systems with enhanced cytotoxicity, selectivity, and mechanistic specificity. This section summarizes recent advances in the design, anticancer activity, and mechanistic insights of cinnamaldehyde-based derivatives, highlighting their potential as lead compounds for anticancer drug development.

**Figure 7:**
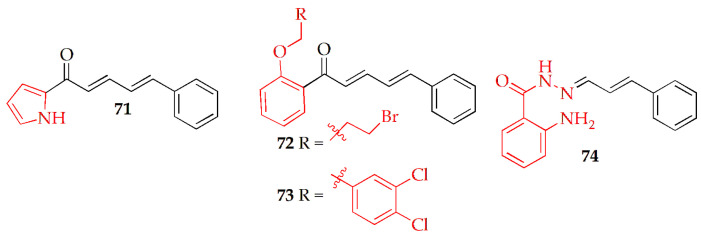
Chemical structures of cinnamaldehyde-based chalcone hybridization (**71**–**73**) and cinnamaldehyde hydrazine (**74**) derivatives.

El-Atawy et al. [107] synthesized a series of cinnamaldehyde-based chalcone derivatives to assess their antioxidant and anticancer potential against human Caco-2 colon cancer cells. These derivatives were synthesized via Claisen–Schmidt condensation of methyl heteroarylketones with substituted cinnamaldehydes in aqueous NaOH/ethanol at room temperature, followed by recrystallization from ethanol to yield yellow solid chalcones Among the tested compounds, compound **71** (Figure 7, Table 4) exhibited the strongest antioxidant activity in the DPPH assay and showed the highest cytotoxicity toward Caco-2 cells, with an IC_50_ of 32.19 ± 3.92 µM, while being non-toxic to normal human lung (Wi38) cells. Treatment of Caco-2 cells with compound **71** significantly increased early and late apoptosis, as confirmed by annexin V/PI and comet assays. Additionally, qRT-PCR and ELISA analyses demonstrated that compound **71** modulated apoptotic gene and protein expression, particularly activating Caspase-3 via the intrinsic apoptosis pathway. These findings suggest that compound **71** holds promise as a potential therapeutic agent for colon cancer.

Kumar et al. [105] synthesized fourteen new cinnamaldehyde–chalcone derivatives and investigated their anticancer, antibacterial, and antifungal activities. The cinnamaldehyde–chalcone derivatives were synthesized via a two-step process involving an aldol condensation of cinnamaldehyde with 2-hydroxyacetophenone to form intermediate 3, followed by alkylation or bromination with substituted benzyl/alkyl halides in DMF/K_2_CO_3_, yielding predominantly monomeric products under mild basic conditions Among these, bromoethane chalcone (**72**) (Figure 7, Table 4) showed the strongest cytotoxicity against DU145, SKBR-3, and HEPG2 cancer cell lines, with IC_50_ values of 8.719, 7.689, and 9.380 mM, respectively, outperforming the other compounds. 2,3-dichlorobenzyl chalcone (**73**) (Figure 7, Table 4) also exhibited notable activity against SKBR-3 and HEPG2 cells. Erythrocyte osmotic fragility assays indicated that **72**, **73** and **73** possess membrane-disruptive properties comparable to quercetin. Molecular docking revealed that **72** binds strongly to succinate dehydrogenase (binding energy −12.9 kcal/mol), surpassing the standard inhibitor malonate (−4.8 kcal/mol). Toxicity studies in mice confirmed **72**’s safety up to 1000 mg/kg with no adverse effects. Overall, compound **72** emerged as a promising and safe lead candidate for further development as an anticancer agent.

Chang et al. [108] synthesized a cinnamaldehyde derivative (**74**) (Figure 7, Table 4) by reacting anthraniloyl hydrazine and cinnamaldehyde in ethanol at 70 °C for 7 h, and after TLC confirmation, solvent removal and washing with dichloromethane afforded the cinnamaldehyde derivative in 95% yield. Using the desolvation method, they formulated BSA–**74** nanoparticles (NPs), which exhibited an increased isoelectric point (5.5–6.0) suitable for tumor microenvironments. Microscopic analysis showed that the nanoparticles were uniformly dispersed, spherical, and approximately 172 nm in size. Spectroscopic characterization indicated enhanced β-turn and antiparallel β-sheet structures, along with a red-shifted fluorescence peak at 370 nm, suggesting improved molecular conjugation after modification. Gel permeation chromatography confirmed that each BSA molecule was conjugated with 56 (**74**) units, giving a molecular weight of 83.629 kDa. The nanoparticles remained stable for up to 30 days at room temperature, unlike free cinnamaldehyde. Cellular uptake studies demonstrated that BSA–73 NPs effectively entered cancer cells and exhibited strong anticancer activity, with a laryngeal cancer inhibition rate exceeding 80%. Overall, the study highlighted BSA–**74** nanoparticles as stable and highly potent anticancer delivery systems.

Across cinnamaldehyde-based derivatives, distinct modification strategies such as chalcone formation via Claisen–Schmidt condensation, halogenated or alkylated chalcone hybrids, and macromolecular nanoparticle conjugation demonstrate varying impacts on anticancer efficacy and selectivity. Chalcone hybridization, as seen in El-Atawy et al. [107], produced compound **71** with moderate cytotoxicity against Caco-2 cells (IC_50_ ≈ 32 µM) alongside strong antioxidant activity and intrinsic apoptosis induction via caspase-3 activation, highlighting the benefit of combining the cinnamaldehyde pharmacophore with an α,β-unsaturated ketone system to enhance redox-modulating and pro-apoptotic effects while maintaining low normal-cell toxicity. In contrast, Kumar et al.’s [105] halogenated chalcone derivatives, particularly bromoethane chalcone (**72**), achieved substantially higher potency (low micromolar IC_50_ values) and strong target engagement with succinate dehydrogenase, suggesting that electron-withdrawing substituents and lipophilicity-enhancing groups can significantly improve cytotoxicity, albeit with a mechanism partly linked to membrane-disruptive properties. Meanwhile, Chang et al. employed a delivery-oriented strategy by conjugating a cinnamaldehyde hydrazone derivative to BSA nanoparticles, markedly improving stability, cellular uptake, and tumor-selective activity (>80% inhibition), demonstrating that nanoformulation can overcome pharmacokinetic and stability limitations of free cinnamaldehyde derivatives. Overall, chalcone hybridization appears most effective for mechanistically driven apoptosis induction, halogenated substitutions enhance potency through increased lipophilicity and target binding, and nanoparticle conjugation offers the greatest translational advantage by improving stability and tumor targeting, indicating that both structural modification and formulation strategies play complementary roles in optimizing anticancer activity and selectivity.

## 5. Comparative Evaluation of Structural Modification Strategies Across EO Scaffolds

Across the five EO-derived scaffolds reviewed (thymol, carvacrol, menthol, eugenol, and cinnamaldehyde), several recurring structural modification strategies have been employed to enhance anticancer activity and selectivity, each showing distinct advantages and limitations. Among these, pharmacophore hybridization and heterocycle incorporation emerged as the most consistently effective approaches. Hybrid molecules combining EO cores with established bioactive motifs (e.g., ciprofloxacin, salicylic acid, or chalcone frameworks) frequently demonstrated improved cytotoxic potency and, in some cases, enhanced selectivity toward cancer cells, likely due to simultaneous engagement of multiple biological targets such as DNA gyrase, MMP-9, or ERα. Similarly, incorporation of heterocyclic linkers particularly triazoles, oxadiazoles, and chalcones, proved highly effective in increasing antiproliferative activity and enabling defined molecular interactions with enzymes such as TS, COX-2, and EGFR. These strategies were especially successful for eugenol- and thymol-based derivatives, where submicromolar to low micromolar IC_50_ values were frequently observed alongside mechanistic evidence of apoptosis induction and cell cycle arrest.

In contrast, simpler derivatization approaches such as esterification, halogenation, and Mannich base formation produced more variable outcomes. While these modifications often improved lipophilicity and membrane permeability, their effects on potency and selectivity were less consistent and appeared highly dependent on the nature and position of substituents within individual compound series. For instance, methoxy substitution or amino-functionalization enhanced activity in certain eugenol derivatives, yet these trends were not universally reproducible across different scaffolds, highlighting the context-dependent nature of SAR conclusions. Metal complexation and porphyrin conjugation represented a distinct strategy aimed at introducing photodynamic or redox-mediated cytotoxic mechanisms; these approaches showed moderate in vitro potency but offered the advantage of multimodal activity, particularly under light-activated conditions. Additionally, nanoparticle conjugation, as demonstrated for cinnamaldehyde derivatives, primarily improved physicochemical stability, cellular uptake, and tumor microenvironment compatibility rather than intrinsically increasing molecular potency, underscoring its role as a delivery-focused rather than purely structural optimization strategy.

Overall, heterocycle incorporation and pharmacophore hybridization appear to be the most promising modification strategies for achieving substantial improvements in anticancer potency and mechanistic specificity, whereas simpler substitutions and linker variations provide incremental but less predictable benefits. Delivery-oriented modifications, including nanoparticle conjugation, offer complementary advantages by addressing pharmacokinetic and stability limitations. However, the relative effectiveness of each strategy varies across scaffold classes and biological targets, and direct cross-study comparisons remain limited by differences in assay conditions, cell models, and selectivity assessments. Consequently, while these modification strategies collectively demonstrate the versatility of EO scaffolds as anticancer lead structures, their optimization requires scaffold-specific and target-oriented design supported by standardized biological evaluation.

## 6. Conclusions and Future Directions

This review highlights recent progress in the structural modification of selected EO components such as thymol, carvacrol, menthol, eugenol, and cinnamaldehyde for potential anticancer applications. Rational derivatization of these natural scaffolds through strategies such as pharmacophore hybridization, heterocycle incorporation, esterification, halogenation, metal complexation, and nanoparticle conjugation has consistently enhanced anticancer potency. Among these, hybridization with bioactive pharmacophores and incorporation of heterocyclic linkers (e.g., triazoles, oxadiazoles, chalcones) most consistently improved cytotoxic potency and enabled engagement with defined molecular targets. Mechanistic studies revealed that these derivatives act through multiple pathways, including mitochondrial apoptosis, cell cycle regulation, and inhibition of key oncogenic targets such as EGFR, PI3K/AKT, thymidylate synthase, VEGFR-2, BRAF, ABL1, DNA gyrase, and MMP-9. SAR analyses underscore the importance of strategic heterocyclic substitution, increased lipophilicity, preservation of key pharmacophores, and hybridization with known bioactive motifs.

However, despite these encouraging advances, the overall evidence base remains heterogeneous and methodologically inconsistent. Reported IC_50_ values are often derived from different assay conditions, cell models, and exposure times which limit the validity of direct cross-study potency comparisons and contribute to variability in selectivity indices. Moreover, mechanistic interpretations often combine in vitro functional data, limited in vivo observations, and purely in silico docking predictions without clear differentiation of evidentiary strength, which may overstate mechanistic certainty. Importantly, the predominance of in vitro cytotoxicity data, coupled with sparse pharmacokinetic, metabolic, and long-term toxicity evaluations, underscores substantial translational gaps that preclude definitive conclusions about clinical applicability. Therefore, while structural derivatization of these natural monoterpenes and phenylpropanoids clearly enhances anticancer potential and provides valuable lead scaffolds, future research should prioritize standardized experimental protocols, rigorous comparative benchmarking within the same biological context, and expanded in vivo and pharmacokinetic validation to more realistically define their therapeutic promise.

## Figures and Tables

**Table 4:** Anticancer activity of selected cinnamaldehyde-based hybrid compounds against human cancer cell lines. IC_50_ values are reported as described in the original studies and should be interpreted within their respective experimental conditions rather than as directly comparable metrics across studies.

Compounds	Evidence Level/Proposed Mechanism	Cell Lines Tested (IC_50_, µM)	Reference Drug/Comparator	Bibliography
**71**	Induces intrinsic apoptosis via caspase-3 activation; selective toward cancer cells	Caco-2 cells (32.19 ± 3.92)	5-Fluorouracil: Caco-2 cells (33.12 ± 1.45)	[107]
**72**	Succinate dehydrogenase inhibition; disrupts energy metabolism and membrane integrity	HEK-293 (20.391 ± 1.6); DU145 (17.861 ± 3.4); SKBR-3 (22.421 ± 2.4); HEPG2 (9.190 ± 0.6)	Doxorubicin: HEK-293 (6.12 ± 05); DU145 (0.45 ± 0.52); SKBR-3 (0.7 ± 0.56); HEPG2 (2.5 ± 1.42)	[105]
**73**		HEK-293 (18.61 ± 1.1); DU145 (8.719 ± 1.8); SKBR-3 (7.689 ± 2.8); HEPG2 (9.380 ± 1.6)		
**74**	BSA–nanoparticle conjugation improves stability, cellular uptake, and intracellular delivery.	NR	NR	[108]

NR = not reported. IC_50_ values were extracted from independent studies conducted under heterogeneous experimental conditions (e.g., assay type, incubation time, cell density, and calculation methods). Therefore, these values should be interpreted within each study’s context and are presented to illustrate activity trends rather than to provide direct cross-study potency rankings.

## Data Availability

No new data were created or analyzed in this study.
